# Targeting STING signaling for the optimal cancer immunotherapy

**DOI:** 10.3389/fimmu.2024.1482738

**Published:** 2024-10-09

**Authors:** Yan Xu, Ying Xiong

**Affiliations:** Department of Obstetrics and Gynecology, Haiyan People’s Hospital, Jiaxing, China

**Keywords:** STING, PD-1, PD-L1, cancer immunotherapy, the tumor microenvironment

## Abstract

Despite the transformative impact of anti-PD-1/PD-L1 therapies, challenges such as low response rates persist. The stimulator of interferon genes (STING) pathway, a crucial element of innate immunity, emerges as a strategic target to overcome these limitations. Understanding its multifaceted functions in cancer, including antigen presentation and response to DNA damage, provides valuable insights. STING agonists, categorized into cyclic dinucleotides (CDNs) and non-CDNs, exhibit promising safety and efficacy profiles. Innovative delivery systems, including antibody-drug conjugates, nanocarriers, and exosome-based therapies, address challenges associated with systemic administration and enhance targeted tumor delivery. Personalized vaccines, such as DT-Exo-STING, showcase the adaptability of STING agonists for individualized treatment. These advancements not only offer new prospects for combination therapies but also pave the way for overcoming resistance mechanisms. This review focuses on the potential of targeting STING pathway to enhance cancer immunotherapy. The integration of STING agonists into cancer immunotherapy holds promise for more effective, personalized, and successful approaches against malignancies, presenting a beacon of hope for the future of cancer treatment.

## 1 Background

In recent years, immunotherapy, with a particular focus on anti-PD-1/PD-L1 antibodies, has emerged as a groundbreaking paradigm in cancer treatment (1, 2). The remarkable advances in this field have revolutionized the therapeutic landscape, harnessing the immune system’s potential to combat malignancies. The introduction of anti-PD-1/PD-L1 therapies has marked a significant stride forward, offering a promising avenue for more effective and targeted cancer interventions (3–11). However, despite the promise and success observed in some cases, the clinical utility of anti-PD-1/PD-L1 therapies faces substantial challenges, primarily characterized by a low response rate among patients (12–14). This limitation underscores the need for a comprehensive understanding of the factors influencing treatment outcomes. The intricacies of the tumor microenvironment (TME) play a critical role in shaping the efficacy of anti-PD-1/PD-L1 therapies (15). Within the TME, a myriad of immunosuppressive factors acts synergistically to impede the optimal function of these therapies (16–19).

Multiple hurdles within the TME contribute to the suboptimal response rates observed with anti-PD-1/PD-L1 antibodies (20). These obstacles include defects in immune checkpoint signaling, the accumulation of immunosuppressive cells, and antigen presentation deficiency (21). The dynamic interplay of these factors underscores the complexity of the TME and its role in modulating tumor progression (22–24). Recognizing the challenges posed by the multifaceted immunosuppressive factors, there is growing interest in exploring novel strategies to enhance the efficacy of immunotherapy. One such promising avenue is the targeting of the stimulator of interferon genes (STING) pathway (25). The STING pathway, an integral component of the innate immune system, is implicated in recognizing cellular stress and infection (26–29). Recent findings suggest that manipulating the STING pathway could offer a means to overcome the limitations associated with anti-PD-1/PD-L1 therapies (30, 31).

Understanding the interplay between immunotherapy and the intricate dynamics of the TME is crucial for advancing cancer treatment strategies (32). This review aims to summarize the advances and limitations of anti-PD-1/PD-L1 immunotherapies, unravel the complexities of immunosuppressive factors within the TME, and explore the potential of targeting the STING pathway as a strategic approach to augment the efficacy of immunotherapy. By elucidating these aspects, we aim to contribute valuable insights for more effective and personalized cancer treatment modalities.

## 2 The cGAS-STING signaling pathway

The cGAS-STING signaling pathway, a prominent cytosolic DNA-sensing mechanism, stands as a cornerstone in the innate immune system, orchestrating responses against pathogens (33–36). Beyond its primary role in pathogen recognition, this pathway intricately regulates a spectrum of cellular functions, spanning from the induction of antiviral interferon responses, cytokine production, autophagy, metabolism, senescence, metastasis to apoptosis (37–45). At the heart of this pathway lies the STING, an endoplasmic reticulum-associated transmembrane protein activated by the endogenous cyclic dinucleotide (CDN) second messenger, 2′3′-cGAMP, produced by the enzyme cGAMP synthase (cGAS) upon binding to cytosolic DNA (46). STING’s activation initiates a cascade involving downstream effectors TANK-binding kinase-1 (TBK1) and IFN regulatory factor-3 (IRF3), resulting in robust innate immune responses (47).

**Figure 1:**
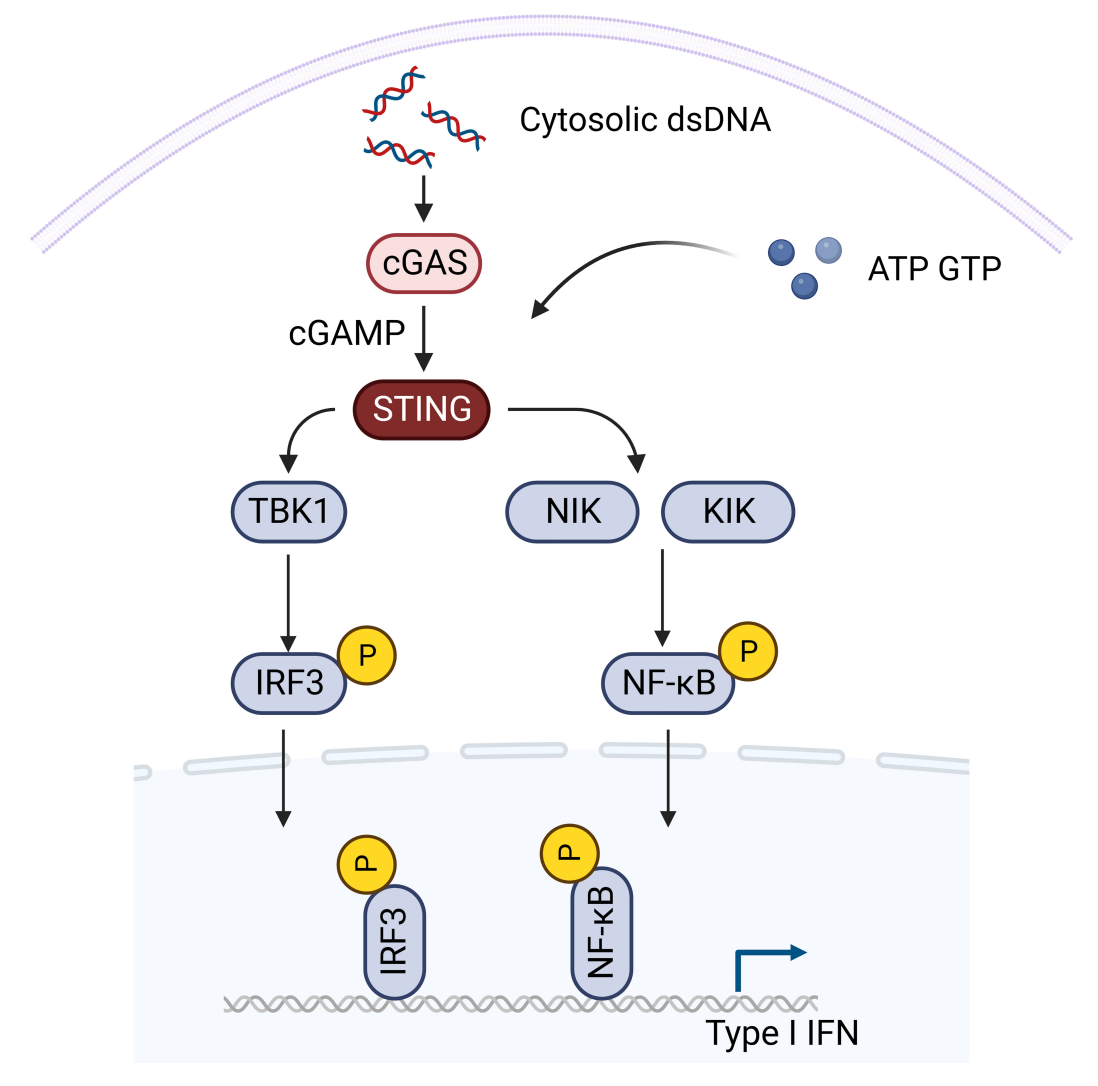
The STING signaling pathway. Activated STING undergoes conformational changes, engaging TBK1 and IRF3. TBK1, forming a homodimer, interacts with two STING molecules, inducing a conformational shift that releases the C-terminal tail of STING. This enables recruitment and activation of TBK1, leading to phosphorylation of serine 366 of the STING C-terminal tail. Phosphorylated IRF3 undergoes homodimerization, translocates to the nucleus, and triggers the transcription of type I interferon (IFN) and other interferon-stimulated genes (ISGs). The STING-TBK1-IRF3 axis acts as a critical link in antiviral defense, orchestrating IFN production to curb viral replication and spread. Importantly, the STING-TBK1-IRF3 pathway also intersects with canonical NF-κB signaling (Created with Biorender).

The STING-TBK1-IRF3 signaling transduction pathway emerges as a pivotal element in the cGAS-STING cascade, translating STING activation into effective immune responses (**Figure 1**). Activated STING undergoes conformational changes, engaging TBK1 and IRF3 (48). TBK1, forming a homodimer, interacts with two STING molecules, inducing a conformational shift that releases the C-terminal tail of STING (49). This enables recruitment and activation of TBK1, leading to phosphorylation of serine 366 of the STING C-terminal tail. Phosphorylated IRF3 undergoes homodimerization, translocates to the nucleus, and triggers the transcription of type I interferon (IFN) and other interferon-stimulated genes (ISGs) (50, 51). The STING-TBK1-IRF3 axis acts as a critical link in antiviral defense, orchestrating IFN production to curb viral replication and spread. Importantly, the STING-TBK1-IRF3 pathway also intersects with canonical NF-κB signaling, involving the NF-κB subunit p65 (52). Although the precise mechanisms of STING-induced NF-κB activation are under investigation, it seems to be independent of ER-to-Golgi trafficking (49). This intricate crosstalk adds another layer of complexity to the diverse cellular functions regulated by the cGAS-STING pathway (53). Therapeutic activation of STING to boost immune responses may inadvertently overactivate NF-κB, leading to harmful inflammation (54). Combining STING agonists with inhibitors of negative regulators in the NF-κB pathway may enhance anti-tumor immunity while controlling inflammation (55). Modulating the crosstalk can help in designing therapies that suppress pathological immune responses without compromising host defense. Understanding individual variations in the STING and NF-κB pathways can aid in predicting patient responses and tailoring treatments. Additionally, STING is involved in extensive crosstalk with various other immune associated signaling pathways, referred as non-canonical STING pathway (40, 56–58). Developing drugs that specifically target components of the crosstalk may provide therapeutic benefits with fewer side effects.

The intricate regulation of the cGAS-STING pathway is paramount, as aberrant activation may lead to severe autoinflammatory or autoimmune diseases (59–61). Recent advances have illuminated the mechanisms of STING activation and the meticulous regulation of this pathway to prevent excessive signaling (62). Furthermore, the cGAS-STING pathway assumes a pivotal role in antimicrobial immunity, influencing responses to diverse pathogens, including viruses and bacteria, thereby shaping immune regulation, and offering potential avenues for therapeutic interventions (63).

## 3 The role of STING signaling in cancer

The STING signaling has been believed to be a central player in the dynamic landscape of cancer immunology, with a particular emphasis on its intricate involvement in cancer antigen presentation (64–66). While the pro-inflammatory role of IFN signaling has fueled interest in STING as a mediator of effective antitumor immunity, recent insights reveal the multifaceted functions of this pathway in cancer, demanding careful contextual consideration (67). A unique feature of cancer is chromosomal instability, marked by accumulated chromosome mis-segregation during mitosis (68). This results in the formation of micronuclei, rupturing during S-phase, exposing genomic double-stranded DNA (dsDNA) to the cytoplasm (69–71). The chronic activation of the cGAS-STING pathway in cancers exhibiting rampant chromosome instability is further compounded by DNA damage induced by radiation therapy, chemotherapeutic agents, and mitochondrial DNA leakage due to oxidative stress (72–78). Nevertheless, tumors exhibit a remarkable ability to regulate the expression of STING pathway genes to evade its antitumor and pro-inflammatory effects. Loss of chromosome 9p, harboring the IFN-gene cluster, is common in certain cancers, allowing these tumors to signal through the NF-κB pathway without inducing IFN response (79). Other evasion mechanisms involve the downregulation of STING levels, observed in various cancers (80).

**Figure 2:**
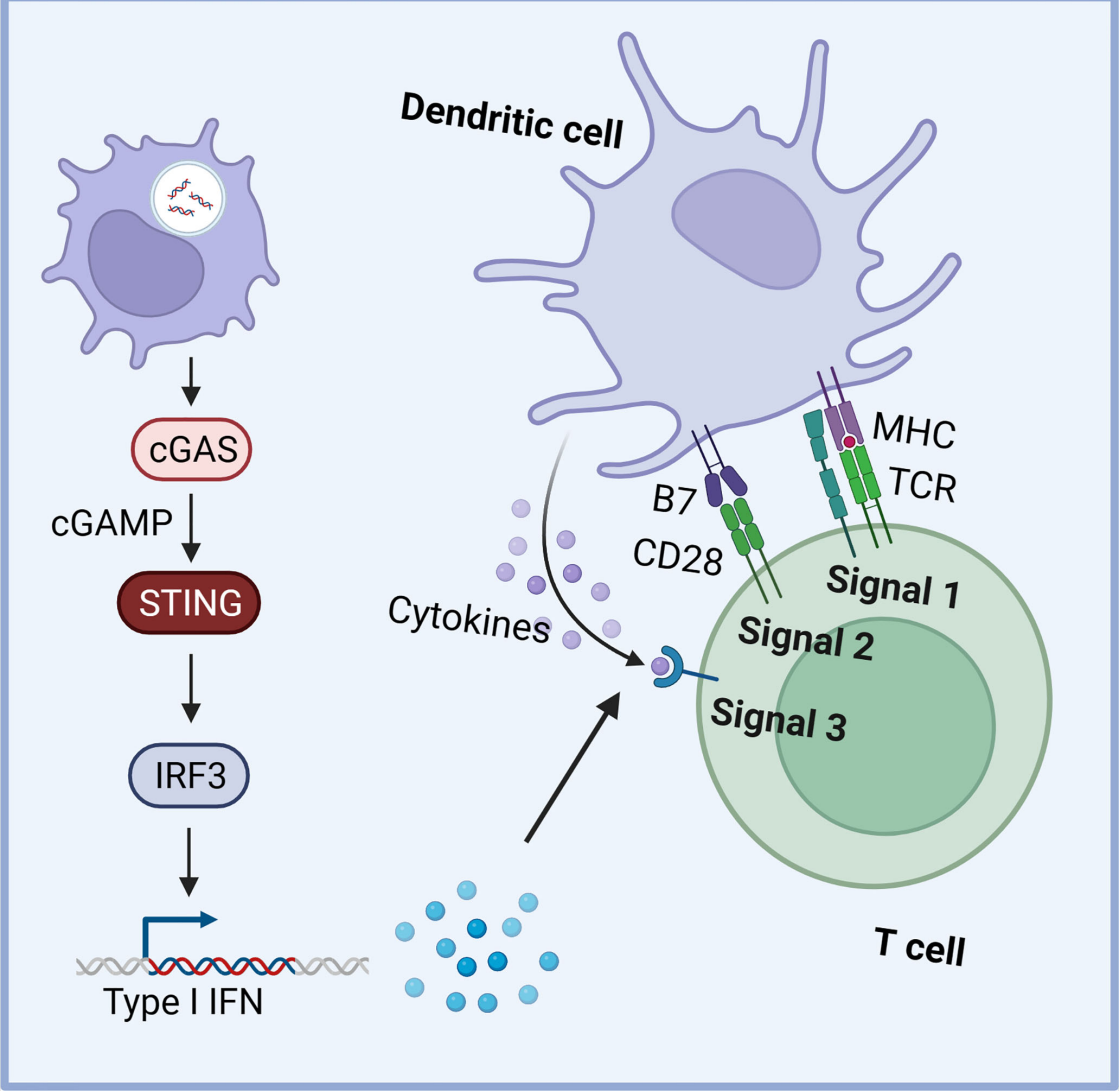
The STING signaling in dendritic cells (DCs). The STING pathway in DCs plays a vital role by taking up tumor-derived DNA, leading to increased type I IFN expression. This enhances DC cross-presentation, survival, lymph node homing, and the expression of Th1 chemokines, crucial for immune cell trafficking (Created with Biorender).

Moreover, tumor-derived cyclic GMP-AMP (cGAMP), a critical activator of the cGAS-STING pathway, can be derived from cancer cells and transferred into neighboring cells, directly activating STING in the TME (81). This transfer occurs through various mechanisms, including import through cell gap junctions, cGAMP importer SLC19A1, connexin, and exosomes (81–84). The delivery of cGAMP or dsDNA to non-tumor cells has been shown to exert both antitumor effects and promote tumor progression, underscoring the intricate interplay between the STING pathway and the TME (49). For instance, the STING pathway in dendritic cells (DCs) plays a vital role by taking up tumor-derived DNA, leading to increased type I IFN expression (85). This enhances DC cross-presentation, survival, lymph node homing, and the expression of Th1 chemokines, which are crucial for immune cell trafficking (**Figure 2**) (86–88). STING inhibition in DCs impairs antigen presentation and reduces tumor-infiltrating lymphocytes (TIL) (74).

Furthermore, tumor-derived cGAMP also plays a pivotal role in natural killer (NK) cell activation. In mouse models, persistent cGAS activation in cancer cells, followed by paracrine cGAMP uptake by neighboring NK cells, enhances IFN-I signaling and antitumor immunity (89). Despite initial enthusiasm for the use of STING agonists in anti-cancer therapy, challenges in translating results from mouse models to humans have surfaced. The efficacy of STING agonists, designed to boost antitumor immunity, has been limited in humans, suggesting that tumors evolve mechanisms to undermine IFN-I signaling (90). These complexities highlight the need for a nuanced understanding of the cGAS-STING pathway in cancer immunotherapy, considering factors such as tumor type, dose-dependent effects, and the dynamic nature of the TME.

## 4 The application of STING agonist in cancer immunotherapy

The evolving field of cancer immunotherapy has witnessed remarkable progress with the emergence of STING agonists, which hold immense potential in harnessing the immune system to combat tumors (91). Here, we summarize the diverse landscape of STING agonists, categorizing them into two groups: CDNs and non-CDNs.

### 4.1 CDNs

The early forays into CDNs introduced compounds like DMXAA and ADU-S100. Despite DMXAA’s limited translational success in human trials due to its weak binding to human STING protein (92), ADU-S100 has emerged as a promising candidate (93–95). In clinical trials, ADU-S100 demonstrated safety and elicited encouraging responses, especially when combined with checkpoint inhibitors. In the phase I trial NCT02675439, ADU-S100 exhibited remarkable safety and tolerability in patients with advanced cancers (96). Despite limited clinical activity as a single agent, noteworthy treatment responses included a partial response in Merkel cell carcinoma and two unconfirmed partial responses in parotid cancer and myxofibrosarcoma (96). Besides, in the phase Ib dose-escalation study NCT03172936, the safety and tolerability of combining ADU-S100 with the PD-1 inhibitor Spartalizumab were evaluated in 106 patients with advanced solid tumors or lymphomas. Administered through weekly intratumoral injections of ADU-S100 and a fixed dose of intravenous spartalizumab (400 mg) every four weeks, the combination demonstrated favorable safety profiles, with common adverse events including pyrexia, injection site pain, and diarrhea (97). Despite minimal antitumor responses (ORR: 10.4%), the study highlighted the feasibility of this combination, even in patients with anti-PD-1 refractory disease, warranting further exploration and optimization in the ongoing quest for effective cancer immunotherapies (97).

Moreover, MK-1454, a synthetic CDN, has showcased notable antitumor activity in phase I trials, particularly when administered in combination with the anti-PD-1 agent pembrolizumab (98, 99). Besides, JNJ-4412, a novel CDN STING agonist, demonstrated efficacy in preclinical models, highlighting its potential through intratumoral injection. The results from these preclinical studies suggest that JNJ-4412 holds promise as a potent STING agonist, potentially contributing to the arsenal of therapeutic options for cancer treatment (100). Furthermore, the exploration of novel STING agonists for systemic delivery unveils a spectrum of promising candidates. SB11285, a small molecule CDN STING agonist, is currently undergoing clinical evaluation for intravenous administration (101, 102). Preclinical models have demonstrated its higher inhibition of tumor growth, hinting at its potential systemic benefits in cancer patients (101). Additionally, Sun et al. present a cancer metalloimmunotherapy prototype employing CDN STING agonists and Mn^2+^ ions assembled into a nanoparticle (CDN-Mn^2+^particle, CMP). This modality administered via either intratumoral injection or systemic intravenous injection, elicited potent anti-tumor immune responses and demonstrated remarkable therapeutic efficacy with abridged doses of STING agonists in various preclinical models (103).

### 4.2 Non-CDN STING agonists

The non-CDN category features innovative compounds with unique structures and mechanisms. SNX281, another small molecule agent, is being investigated in a phase I dose escalation study for advanced solid tumors (104, 105). Also, BMS-986301, exhibiting robust antitumor efficacy pre-clinically, has transitioned into clinical trials, reflecting its potential as an effective systemic STING agonist (106). The development of CRD-5500 showcases its effectiveness through both intravenous and intratumoral routes in murine models. This versatility positions CRD-5500 as a favorable candidate for future clinical development, offering flexibility in therapeutic administration (104, 107). Moreover, TTI-10001 has demonstrated safety and antitumor activity in preclinical models (108). Besides, ALG-031048 exhibited higher stability compared to ADU-S100 and demonstrated significant tumor regression in preclinical models. Its dose-dependent increase in cytokine levels and enhanced antitumor efficacy, in combination with anti-PD1 therapy position ALG-031048, make it a promising candidate for further clinical exploration (109).

Notably, some novel non-CDN compounds exhibit potential for systemic delivery, with enhanced stability, opening avenues for broader application in cancer immunotherapy. For example, identified through a screen for IFN-β secretion inducers, MSA-2 operates through a unique mechanism, binding to both human and mouse STING as a noncovalent dimer. Administered orally or subcutaneously, MSA-2 induced elevations of interferon-β in plasma and tumors, demonstrating well-tolerated regimens and prompting tumor regressions in mice with MC38 syngeneic tumors (110). Structural analyses unveiled MSA-2’s binding as a noncovalent dimer to STING in a “closed-lid” conformation, shedding light on its mechanism of action. MSA-2’s ability to preferentially activate STING in tumors positions it as a promising candidate for developing human STING agonists that are amenable to systemic administration in patients (110). In the later preclinical studies, MSA-2 synergized with other immunotherapies, such as anti-TGF-β/PD-L1 bispecific antibodies, to overcome immunotherapy resistance in murine tumor models (111, 112). Also, unlike current efforts focused on modified cyclic dinucleotides for intratumoral delivery, ABZI demonstrated systemic efficacy in treating tumors (113). Developed through a linking strategy, di-ABZIs exhibit enhanced binding to STING and cellular function (113). Intravenous injection of di-ABZI STING agonist in mice with syngeneic colon tumors resulted in potent antitumor activity, leading to complete and lasting tumor regression (113). This milestone marks a significant advancement in the quest for effective immune-modulating cancer treatments (113). Additionally, JNJ-6196 also demonstrated systemic efficacy, positioning JNJ-6196 as a compelling candidate for clinical development (114).

### 4.3 STING agonists with novel delivery systems

To overcome safety concerns with systemic STING agonist administration and limited accessibility with intratumoral injection, antibody-drug conjugates (ADCs) were developed. These ADCs, combining a STING agonist with tumor-targeting antibodies, demonstrated well-tolerated systemic administration and potent antitumor efficacy in mouse models. For instance, the anti-EGFR-172 ADC demonstrates the feasibility of delivering a STING agonist selectively to tumors (115). The adaptability of IMSA172 for conjugation with various antibodies and tumor-targeting agents opens avenues for exploring different ADCs, unveiling their safety and efficacy in activating STING across diverse tumor types through systemic delivery (115). Besides, Duvall et al. developed a STING agonist ADC platform to address the translational challenges of STING agonists in the clinic (116). This platform, featuring a potent non-cyclic dinucleotide STING agonist, a cleavable ester-based linker, and a hydrophilic PEG8-bisglucamine scaffold, exhibits robust and durable antitumor activity, high stability, and favorable pharmacokinetics in nonclinical species, showcasing its potential for systemic administration and localized STING activation within tumors for enhanced therapeutic efficacy and tolerability (116).

Furthermore, various nanocarriers are promising for the delivery of STING agonists. Zhou developed a nanovaccine to resolve the difficulty in delivering mRNA and nucleic acid drugs. The nanovaccine demonstrated the activation of potent antitumor immune response and long-term immune memory by transferring mRNA antigen and cGAMP (117). Optimization of key parameters, such as modifying the PBA moiety and utilizing the anionic Lipo-ORG for lymphatic delivery, resulted in suppressed tumor growth and metastasis, extended survival, and synergistic effects with PD-L1 blockade in a B16-OVA tumor model (117). Besides, some novel pH-responsive DNA nanovaccines, featuring PLA-b-PEG in the core and pH-responsive i-motif DNA on the surface, efficiently load and release CDG in immune cell endosomes, promoting potent antitumor immune responses, overcoming immunosuppression, and demonstrating superior efficacy in a murine melanoma model compared to liposomal CDG and fluoride-CDG (118). In addition, Gu et al. designed a novel antigen-inspired MnO2 nanovaccine, serving as a Mn^2+^ source and functionalized with mannose for specific delivery to innate immune cells (119). This nanovaccine activated the STING pathway, enhancing radiotherapy-induced immune responses and inhibiting both local and distant tumors, while also allowing for magnetic resonance imaging to monitor *in vivo* distribution (119). Actually, there are many STING-activating cancer vaccines exhibiting potent antitumor activity in preclinical studies, including STINGVAX, CDN/neoantigen co-delivering nanovaccines, PC7A, and self-degradable poly(β-amino ester)s (120–123).

Finally, exosome-based therapies leverage cell-derived nanovesicles to deliver STING agonists to tumors. Cheng et al. designed multifunctional hybrid exosomes to activate the cGAS-STING pathway (124). These exosomes were created by merging genetically engineered exosomes carrying CD47 from tumor cells with those from M1 macrophages, encapsulating them with a DNA-targeting agent (SN38) and a STING agonist (MnO2) (124). The hybrid exosomes exhibited excellent tumor-targeting capabilities and prolonged circulation time, inducing polarization of tumor-associated macrophages to the M1 phenotype, releasing SN38 to cause DNA damage, and stimulating cGAS/STING activation with Mn^2+^ at the tumor site. This multifunctional approach promoted DC maturation, facilitated cytotoxic T lymphocyte infiltration, and recruited natural killer cells to the tumor region, resulting in significant antitumor and antimetastatic efficacy (124). Notably, Liu et al. explored Artemisia annua, a plant recognized for its anti-malarial properties, and isolated exosome-like particles termed artemisia-derived nanovesicles (ADNVs) (125). These nano-scaled vesicles exhibited the remarkable ability to inhibit tumor growth and enhance antitumor immunity in a lung cancer mouse model. The key player identified within these vesicles was plant-derived mitochondrial DNA (mtDNA), which, upon internalization by tumor-associated macrophages, activated the cGAS-STING pathway, shifting pro-tumor macrophages to an antitumor phenotype (125). Additionally, administration of ADNVs significantly improved the effectiveness of a PD-L1 inhibitor, showcasing the potential of this inter-kingdom interaction to stimulate immunostimulatory signaling and bolster antitumor immunity (125). Besides, in the work of Bao et al., DC-tumor hybrid cell-derived chimeric exosomes loaded with STING agonists (DT-Exo-STING) were engineered to address the challenge of balancing antigen-enriched delivery and optimal antigen-presentation functionality in DCs (126). These chimeric carriers, equipped with broad-spectrum antigen complexes, induce a potent T-cell response through both direct self-presentation and indirect DC-to-T immune stimulation (126). The nanovaccine-driven STING activation not only surpassed conventional CDN delivery methods in tissue-homing capacity, including penetration of the blood-brain barrier, but also ensured efficient cytosolic entry for activating STING signaling (126). This strategy not only improves antigen presentation but also transforms immunosuppressive TME into a pro-inflammatory state, resulting in a significant reduction of intracranial primary lesions (126). Moreover, the personalized DT-Exo-STING vaccines, utilizing autologous tumor tissues, enhance sensitivity to ICB and establish systemic immune memory against cancer recurrence. These findings offer a promising avenue for glioblastoma immunotherapy, with potential implications for further exploration in clinical applications (126).

However, as alluded earlier, the potent activation of the innate immune system by using STING agonists can lead to several side effects that are critical to consider in clinical settings. STING activation leads to the production of type I interferons and pro-inflammatory cytokines. While beneficial in fighting tumors and infections, this can result in systemic inflammation, causing symptoms like fever, chills, and fatigue. Overstimulation of the immune system may also trigger autoimmune reactions. The heightened immune activity can cause the body to attack its own tissues, potentially leading to conditions such as lupus or rheumatoid arthritis. Furthermore, STING agonists occasionally elicit Cytokine Release Syndrome (CRS), also known as a “cytokine storm,” is a severe immune reaction characterized by the rapid release of large amounts of cytokines (127). This can lead to organ dysfunction and is a serious concern with immunotherapies. Hence, reaching the next milestone for oncologists is to concurrently diminish and even eliminate side effects, while enhancing immune efficiency, prolonging lifespan, and improving patients’ quality of life.

## 5 Perspective and conclusion

In conclusion, the landscape of cancer immunotherapy has evolved significantly with the emergence of STING signaling as a promising target. The limitations associated with anti-PD-1/PD-L1 therapies, including low response rates attributed to the intricate dynamics of the TME, have prompted exploration into novel strategies. The STING pathway, a central player in innate immunity, presents a unique opportunity to meet these challenges and improve immunotherapy efficacy. Understanding the role of the STING pathway in cancer immunology has revealed its intricate involvement in cancer antigen presentation, response to DNA damage, and modulation of the TME. Despite the complexities and evasion mechanisms exhibited by tumors, targeting the STING pathway holds the potential to influence diverse aspects of the immune attack against cancer.

The application of STING agonists in cancer immunotherapy has seen remarkable progress, with a diverse landscape of CDNs and non-CDNs. CDNs, such as ADU-S100, MK-1454, and JNJ-4412, have demonstrated safety and efficacy in clinical trials, particularly when combined with checkpoint inhibitors (**Table 1**). Non-CDN STING agonists, like MSA-2 and di-ABZI, showcase unique structures and mechanisms, expanding the options for therapeutic interventions. Moreover, the development of STING agonists with novel delivery systems, including ADCs, nanocarriers, and exosome-based therapies, addresses safety concerns and enhances the potential for systemic administration. These innovative approaches demonstrate the adaptability of STING agonists for selective tumor targeting, promoting antitumor immune responses, and overcoming challenges in drug delivery. These advancements open avenues for exploring combination therapies, overcoming resistance mechanisms, and improving the overall efficacy of cancer immunotherapy. In spite of the generalized advertisement in scale of immunotherapy, presenting problems, drawbacks and frustrations still thwart their feasibility and accessibility to fide bona clinical employment. In summary, pharmacokinetic challenges, escalating adverse effects, suppressive tumor microenvironment through alternative pathways, and genetic variability individually or collectively contribute to the current unfavorable state (76, 128). More efforts are warranted to circumvent these hurdles. Furthermore, the implementation of combinative treatment strategies involving STING agonists and other antitumor agents, such as PARP inhibitors and chemotherapies other than ICBs represents a novel endeavor to overcome cancer (129, 130).

**Table 1:** The ongoing clinical using STING agonists in cancer immunotherapy regimen.

Agents Terming	NCT numbers	Indications	Phase	Cardinal observation indications	Schedule and Outcomes
KL340399	NCT05549804/NCT05387928	Advanced Solid Tumors	1	Tolerability and RP2D	Not referred
E7766	NCT04109092	Urinary Bladder Neoplasms	1	Tolerability and CRR	Not referred
	NCT04144140	Lymphoma, Advanced Solid Tumors	1	Tolerability and ORR	Not referred
MIW815/ADU-S100	NCT03937141	Head and Neck Cancer	2	ORR	Intratumoral injection (800 μg per lesion day 1 and 8 of a 21-days cycle) + Pembrolizumab; 4/8 reaching PR; 1/8 reaching SD; 3/8 reaching PD
	NCT02675439	Lymphoma, Advanced/Metastatic Solid Tumors	1	Tolerability and RP2D	Intratumoral injection (50 to 6,400 μg weekly, on a 3-weeks-on/1-week-off schedule); 94% of lesions reaching stable or decrease with systemic immune activity
	NCT03172936	Lymphoma, Advanced/Metastatic Solid Tumors	1	Tolerability	Intratumoral injection (50-800 µg) either weekly (3 weeks on/1 week off) or Q4W + PDR001; The regimen being tolerable; ORR=10.4%
MK-1454	NCT04220866	Head and Neck Cancer	2	ORR	Intratumoral injection (540 ug on Day 1 of every week for two 3-week then on Day 1 of each 3-week cycle for up 33 cycles + Pembrolizumab; ORR = 50%; PFS = 6.4 months
	NCT03010176	Lymphoma, Advanced/Metastatic Solid Tumors	1	Tolerability	Intratumoral injection (10-3000 ug, and 90-1500 ug Q1W*9 for 3cycles and beyond for up to 35 cycles for Arm 1, and 2 respectively), Arm 2 was combined with Pembrolizumab; Regimen being tolerable and PR = 24% (Arm 2), 0 (Arm 1); DCR = 48% (Arm 2), 20% (Arm 1)
CRD3874-SI	NCT06021626	Sarcoma, Merkel Cell Carcinoma	1	Tolerability and ORR	Not referred
TXN10128	NCT05978492	Solid Tumors	1	Tolerability	Not referred
GSK3745417	NCT03843359	Solid Tumors	1	Tolerability	Not referred
	NCT05424380	Hematologic malignancies	1	Tolerability and ORR	Not referred
IMSA101	NCT05846659/NCT05846646	Solid Tumors	2	Progression-free rate	Not referred
SNX281	NCT04609579	Lymphoma, Advanced Solid Tumors	1	Tolerability and RP2D	Not referred
ONM-501	NCT06022029	Lymphoma, Advanced Solid Tumors	1	Tolerability	Not referred
TAK-500	NCT05070247	Solid Tumors	1	Tolerability and ORR	Not referred

RP2D, Recommended Phase 2 Dose; CRR, Complete Response Rate; ORR, Objective Response Rate; PR, Partial Response; SD, Stable Disease; PD, Progression Disease.

In summary, targeting the STING signaling pathway represents a promising strategy for optimizing cancer immunotherapy. As research in this field continues to unravel the complexities of the STING pathway and its interactions within the TME, the potential for innovative and effective treatment modalities grows. The integration of STING agonists into the evolving landscape of cancer immunotherapy offers hope for more personalized and successful approaches to combat malignancies.

## References

[1] Gong J Chehrazi-Raffle A Reddi S Salgia R. Development of PD-1 and PD-L1 inhibitors as a form of cancer immunotherapy: a comprehensive review of registration trials and future considerations. J Immunother Cancer. (2018) 6:8. doi: 10.1186/s40425-018-0316-z 29357948 PMC5778665

[2] Raval RR Sharabi AB Walker AJ Drake CG Sharma P. Tumor immunology and cancer immunotherapy: summary of the 2013 SITC primer. J Immunother Cancer. (2014) 2:14. doi: 10.1186/2051-1426-2-14 24883190 PMC4039332

[3] Cercek A Lumish M Sinopoli J Weiss J Shia J Lamendola-Essel M. PD-1 blockade in mismatch repair-deficient, locally advanced rectal cancer. N Engl J Med. (2022) 386:2363–76. doi: 10.1056/NEJMoa2201445 PMC9492301 35660797

[4] Topalian SL Hodi FS Brahmer JR Gettinger SN Smith DC McDermott DF. Safety, activity, and immune correlates of anti-PD-1 antibody in cancer. N Engl J Med. (2012) 366:2443–54. doi: 10.1056/NEJMoa1200690 PMC3544539 22658127

[5] Olson DJ Eroglu Z Brockstein B Poklepovic AS Bajaj M Babu S. Pembrolizumab plus ipilimumab following anti-PD-1/L1 failure in melanoma. J Clin Oncol. (2021) 39:2647–55. doi: 10.1200/jco.21.00079 PMC8376314 33945288

[6] VanderWalde A Bellasea SL Kendra KL Khushalani NI Campbell KM Scumpia PO. Ipilimumab with or without nivolumab in PD-1 or PD-L1 blockade refractory metastatic melanoma: a randomized phase 2 trial. Nat Med. (2023) 29:2278–85. doi: 10.1038/s41591-023-02498-y PMC10708907 37592104

[7] Ansell SM Lesokhin AM Borrello I Halwani A Scott EC Gutierrez M. PD-1 blockade with nivolumab in relapsed or refractory Hodgkin’s lymphoma. N Engl J Med. (2015) 372:311–9. doi: 10.1056/NEJMoa1411087 PMC4348009 25482239

[8] Hamid O Robert C Daud A Hodi FS Hwu WJ Kefford R. Safety and tumor responses with lambrolizumab (anti-PD-1) in melanoma. N Engl J Med. (2013) 369:134–44. doi: 10.1056/NEJMoa1305133 PMC4126516 23724846

[9] Shi J Liu J Tu X Li B Tong Z Wang T. Single-cell immune signature for detecting early-stage HCC and early assessing anti-PD-1 immunotherapy efficacy. J Immunother Cancer. (2022) 10:e003133. doi: 10.1136/jitc-2021-003133 35101942 PMC8804705

[10] Zhang W Tong S Hu B Wan T Tang H Zhao F. Lenvatinib plus anti-PD-1 antibodies as conversion therapy for patients with unresectable intermediate-advanced hepatocellular carcinoma: a single-arm, phase II trial. J Immunother Cancer. (2023) 11:e007366. doi: 10.1136/jitc-2023-007366 37730273 PMC10514649

[11] Lin Z Cai M Zhang P Li G Liu T Li X. Phase II, single-arm trial of preoperative short-course radiotherapy followed by chemotherapy and camrelizumab in locally advanced rectal cancer. J Immunother Cancer. (2021) 9:e003554. doi: 10.1136/jitc-2021-003554 34725214 PMC8562535

[12] Kwon M An M Klempner SJ Lee H Kim KM Sa JK. Determinants of response and intrinsic resistance to PD-1 blockade in microsatellite instability-high gastric cancer. Cancer Discovery. (2021) 11:2168–85. doi: 10.1158/2159-8290.Cd-21-0219 33846173

[13] Ribas A Medina T Kirkwood JM Zakharia Y Gonzalez R Davar D. Overcoming PD-1 blockade resistance with cpG-A toll-like receptor 9 agonist vidutolimod in patients with metastatic melanoma. Cancer Discovery. (2021) 11:2998–3007. doi: 10.1158/2159-8290.Cd-21-0425 34326162 PMC8799774

[14] Kluger HM Tawbi HA Ascierto ML Bowden M Callahan MK Cha E. Defining tumor resistance to PD-1 pathway blockade: recommendations from the first meeting of the SITC Immunotherapy Resistance Taskforce. J Immunother Cancer. (2020) 8:e000398. doi: 10.1136/jitc-2019-000398 32238470 PMC7174063

[15] Liu X Hogg GD DeNardo DG. Rethinking immune checkpoint blockade: ‘Beyond the T cell’. J Immunother Cancer. (2021) 9:e001460. doi: 10.1136/jitc-2020-001460 33468555 PMC7817791

[16] Pu Y Ji Q. Tumor-associated macrophages regulate PD-1/PD-L1 immunosuppression. Front Immunol. (2022) 13:874589. doi: 10.3389/fimmu.2022.874589 35592338 PMC9110638

[17] Fukumura D Kloepper J Amoozgar Z Duda DG Jain RK. Enhancing cancer immunotherapy using antiangiogenics: opportunities and challenges. Nat Rev Clin Oncol. (2018) 15:325–40. doi: 10.1038/nrclinonc.2018.29 PMC5921900 29508855

[18] Falcomatà C Bärthel S Schneider G Rad R Schmidt-Supprian M Saur D. Context-specific determinants of the immunosuppressive tumor microenvironment in pancreatic cancer. Cancer Discovery. (2023) 13:278–97. doi: 10.1158/2159-8290.Cd-22-0876 PMC9900325 36622087

[19] Genova C Dellepiane C Carrega P Sommariva S Ferlazzo G Pronzato P. Therapeutic implications of tumor microenvironment in lung cancer: focus on immune checkpoint blockade. Front Immunol. (2021) 12:799455. doi: 10.3389/fimmu.2021.799455 35069581 PMC8777268

[20] Pitt JM Marabelle A Eggermont A Soria JC Kroemer G Zitvogel L. Targeting the tumor microenvironment: removing obstruction to anticancer immune responses and immunotherapy. Ann Oncol. (2016) 27:1482–92. doi: 10.1093/annonc/mdw168 27069014

[21] Vitale I Manic G Coussens LM Kroemer G Galluzzi L. Macrophages and metabolism in the tumor microenvironment. Cell Metab. (2019) 30:36–50. doi: 10.1016/j.cmet.2019.06.001 31269428

[22] Ren X Zhang L Zhang Y Li Z Siemers N Zhang Z. Insights gained from single-cell analysis of immune cells in the tumor microenvironment. Annu Rev Immunol. (2021) 39:583–609. doi: 10.1146/annurev-immunol-110519-071134 33637019

[23] Gao X Sui H Zhao S Gao X Su Y Qu P. Immunotherapy targeting myeloid-derived suppressor cells (MDSCs) in tumor microenvironment. Front Immunol. (2020) 11:585214. doi: 10.3389/fimmu.2020.585214 33613512 PMC7889583

[24] Barnestein R Galland L Kalfeist L Ghiringhelli F Ladoire S Limagne E. Immunosuppressive tumor microenvironment modulation by chemotherapies and targeted therapies to enhance immunotherapy effectiveness. Oncoimmunology. (2022) 11:2120676. doi: 10.1080/2162402x.2022.2120676 36117524 PMC9481153

[25] Samson N Ablasser A. The cGAS-STING pathway and cancer. Nat Cancer. (2022) 3:1452–63. doi: 10.1038/s43018-022-00468-w 36510011

[26] Liu N Pang X Zhang H Ji P. The cGAS-STING pathway in bacterial infection and bacterial immunity. Front Immunol. (2021) 12:814709. doi: 10.3389/fimmu.2021.814709 35095914 PMC8793285

[27] Lu Q Chen Y Li J Zhu F Zheng Z. Crosstalk between cGAS-STING pathway and autophagy in cancer immunity. Front Immunol. (2023) 14:1139595. doi: 10.3389/fimmu.2023.1139595 36936940 PMC10014609

[28] Wan D Jiang W Hao J. Research advances in how the cGAS-STING pathway controls the cellular inflammatory response. Front Immunol. (2020) 11:615. doi: 10.3389/fimmu.2020.00615 32411126 PMC7198750

[29] Su T Zhang Y Valerie K Wang XY Lin S Zhu G. STING activation in cancer immunotherapy. Theranostics. (2019) 9:7759–71. doi: 10.7150/thno.37574 PMC6831454 31695799

[30] Jiang M Chen P Wang L Li W Chen B Liu Y. cGAS-STING, an important pathway in cancer immunotherapy. J Hematol Oncol. (2020) 13:81. doi: 10.1186/s13045-020-00916-z 32571374 PMC7310007

[31] Tian Z Zeng Y Peng Y Liu J Wu F. Cancer immunotherapy strategies that target the cGAS-STING pathway. Front Immunol. (2022) 13:996663. doi: 10.3389/fimmu.2022.996663 36353640 PMC9639746

[32] Elhanani O Ben-Uri R Keren L. Spatial profiling technologies illuminate the tumor microenvironment. Cancer Cell. (2023) 41:404–20. doi: 10.1016/j.ccell.2023.01.010 36800999

[33] Domizio JD Gulen MF Saidoune F Thacker VV Yatim A Sharma K. The cGAS-STING pathway drives type I IFN immunopathology in COVID-19. Nature. (2022) 603:145–51. doi: 10.1038/s41586-022-04421-w PMC8891013 35045565

[34] Alonso Paiva IM AS R Brito CB Ferrero MC Ortiz Wilczyñski JM Carrera Silva EA. Role of the cGAS/STING pathway in the control of Brucella abortus infection acquired through the respiratory route. Front Immunol. (2023) 14:1116811. doi: 10.3389/fimmu.2023.1116811 37261352 PMC10227575

[35] Jin X Wang W Zhao X Jiang W Shao Q Chen Z. The battle between the innate immune cGAS-STING signaling pathway and human herpesvirus infection. Front Immunol. (2023) 14:1235590. doi: 10.3389/fimmu.2023.1235590 37600809 PMC10433641

[36] Zhang X Bai XC Chen ZJ. Structures and mechanisms in the cGAS-STING innate immunity pathway. Immunity. (2020) 53:43–53. doi: 10.1016/j.immuni.2020.05.013 32668227

[37] Sun L Wu J Du F Chen X Chen ZJ. Cyclic GMP-AMP synthase is a cytosolic DNA sensor that activates the type I interferon pathway. Science. (2013) 339:786–91. doi: 10.1126/science.1232458 PMC3863629 23258413

[38] Zhu H Zhang R Yi L Tang YD Zheng C. UNC93B1 attenuates the cGAS-STING signaling pathway by targeting STING for autophagy-lysosome degradation. J Med Virol. (2022) 94:4490–501. doi: 10.1002/jmv.27860 35577759

[39] Gui X Yang H Li T Tan X Shi P Li M. Autophagy induction via STING trafficking is a primordial function of the cGAS pathway. Nature. (2019) 567:262–6. doi: 10.1038/s41586-019-1006-9 PMC9417302 30842662

[40] Zhang D Liu Y Zhu Y Zhang Q Guan H Liu S. A non-canonical cGAS-STING-PERK pathway facilitates the translational program critical for senescence and organ fibrosis. Nat Cell Biol. (2022) 24:766–82. doi: 10.1038/s41556-022-00894-z 35501370

[41] Xu D Tian Y Xia Q Ke B. The cGAS-STING pathway: novel perspectives in liver diseases. Front Immunol. (2021) 12:682736. doi: 10.3389/fimmu.2021.682736 33995425 PMC8117096

[42] Gulen MF Samson N Keller A Schwabenland M Liu C Glück S. cGAS-STING drives ageing-related inflammation and neurodegeneration. Nature. (2023) 620:374–80. doi: 10.1038/s41586-023-06373-1 PMC10412454 37532932

[43] Victorelli S Salmonowicz H Chapman J Martini H Vizioli MG Riley JS. Apoptotic stress causes mtDNA release during senescence and drives the SASP. Nature. (2023) 622:627–36. doi: 10.1038/s41586-023-06621-4 PMC10584674 37821702

[44] Hu J Sánchez-Rivera FJ Wang Z Johnson GN Ho YJ Ganesh K. STING inhibits the reactivation of dormant metastasis in lung adenocarcinoma. Nature. (2023) 616:806–13. doi: 10.1038/s41586-023-05880-5 PMC10569211 36991128

[45] Guo Q Chen X Chen J Zheng G Xie C Wu H. STING promotes senescence, apoptosis, and extracellular matrix degradation in osteoarthritis via the NF-κB signaling pathway. Cell Death Dis. (2021) 12:13. doi: 10.1038/s41419-020-03341-9 33414452 PMC7791051

[46] Chen C Xu P. Cellular functions of cGAS-STING signaling. Trends Cell Biol. (2023) 33:630–48. doi: 10.1016/j.tcb.2022.11.001 36437149

[47] Sun Z Hornung V. cGAS-STING signaling. Curr Biol. (2022) 32:R730–r734. doi: 10.1016/j.cub.2022.05.027 35820380

[48] Pan J Fei CJ Hu Y Wu XY Nie L Chen J. Current understanding of the cGAS-STING signaling pathway: Structure, regulatory mechanisms, and related diseases. Zool Res. (2023) 44:183–218. doi: 10.24272/j.issn.2095-8137.2022.464 36579404 PMC9841179

[49] Chauvin SD Stinson WA Platt DJ Poddar S Miner JJ. Regulation of cGAS and STING signaling during inflammation and infection. J Biol Chem. (2023) 299:104866. doi: 10.1016/j.jbc.2023.104866 37247757 PMC10316007

[50] Tanaka Y Chen ZJ. STING specifies IRF3 phosphorylation by TBK1 in the cytosolic DNA signaling pathway. Sci Signal. (2012) 5:ra20. doi: 10.1126/scisignal.2002521 22394562 PMC3549669

[51] Liu S Cai X Wu J Cong Q Chen X Li T. Phosphorylation of innate immune adaptor proteins MAVS, STING, and TRIF induces IRF3 activation. Science. (2015) 347:aaa2630. doi: 10.1126/science.aaa2630 25636800

[52] Dunphy G Flannery SM Almine JF Connolly DJ Paulus C Jønsson KL. Non-canonical activation of the DNA sensing adaptor STING by ATM and IFI16 mediates NF-κB signaling after nuclear DNA damage. Mol Cell. (2018) 71:745–760.e745. doi: 10.1016/j.molcel.2018.07.034 30193098 PMC6127031

[53] Vashi N Bakhoum SF. The evolution of STING signaling and its involvement in cancer. Trends Biochem Sci. (2021) 46:446–60. doi: 10.1016/j.tibs.2020.12.010 PMC8122033 33461879

[54] Messaoud-Nacer Y Culerier E Rose S Maillet I Rouxel N Briault S. STING agonist diABZI induces PANoptosis and DNA mediated acute respiratory distress syndrome (ARDS). Cell Death Dis. (2022) 13:269. doi: 10.1038/s41419-022-04664-5 35338116 PMC8953969

[55] Liu X Hogg GD Zuo C Borcherding NC Baer JM Lander VE. Context-dependent activation of STING-interferon signaling by CD11b agonists enhances anti-tumor immunity. Cancer Cell. (2023) 41:1073–1090.e1012. doi: 10.1016/j.ccell.2023.04.018 37236195 PMC10281762

[56] Zhao H Wu L Yan G Chen Y Zhou M Wu Y. Inflammation and tumor progression: signaling pathways and targeted intervention. Signal Transduct Target Ther. (2021) 6:263. doi: 10.1038/s41392-021-00658-5 34248142 PMC8273155

[57] Ghosh M Saha S Li J Montrose DC Martinez LA. p53 engages the cGAS/STING cytosolic DNA sensing pathway for tumor suppression. Mol Cell. (2023) 83:266–280.e266. doi: 10.1016/j.molcel.2022.12.023 36638783 PMC9993620

[58] Meibers HE Warrick KA VonHandorf A Vallez CN Kawarizadeh K Saha I. Effector memory T cells induce innate inflammation by triggering DNA damage and a non-canonical STING pathway in dendritic cells. Cell Rep. (2023) 42:113180. doi: 10.1016/j.celrep.2023.113180 37794597 PMC10654673

[59] Decout A Katz JD Venkatraman S Ablasser A. The cGAS-STING pathway as a therapeutic target in inflammatory diseases. Nat Rev Immunol. (2021) 21:548–69. doi: 10.1038/s41577-021-00524-z PMC8029610 33833439

[60] Skopelja-Gardner S An J Elkon KB. Role of the cGAS-STING pathway in systemic and organ-specific diseases. Nat Rev Nephrol. (2022) 18:558–72. doi: 10.1038/s41581-022-00589-6 PMC9214686 35732833

[61] Kato Y Park J Takamatsu H Konaka H Aoki W Aburaya S. Apoptosis-derived membrane vesicles drive the cGAS-STING pathway and enhance type I IFN production in systemic lupus erythematosus. Ann Rheum Dis. (2018) 77:1507–15. doi: 10.1136/annrheumdis-2018-212988 PMC6161667 29945921

[62] Konno H Konno K Barber GN. Cyclic dinucleotides trigger ULK1 (ATG1) phosphorylation of STING to prevent sustained innate immune signaling. Cell. (2013) 155:688–98. doi: 10.1016/j.cell.2013.09.049 PMC3881181 24119841

[63] Wang Y Luo J Alu A Han X Wei Y Wei X. cGAS-STING pathway in cancer biotherapy. Mol Cancer. (2020) 19:136. doi: 10.1186/s12943-020-01247-w 32887628 PMC7472700

[64] Lv M Chen M Zhang R Zhang W Wang C Zhang Y. Manganese is critical for antitumor immune responses via cGAS-STING and improves the efficacy of clinical immunotherapy. Cell Res. (2020) 30:966–79. doi: 10.1038/s41422-020-00395-4 PMC7785004 32839553

[65] Graham PT Nowak AK Cornwall SMJ Larma I Nelson DJ. The STING agonist, DMXAA, reduces tumor vessels and enhances mesothelioma tumor antigen presentation yet blunts cytotoxic T cell function in a murine model. Front Immunol. (2022) 13:969678. doi: 10.3389/fimmu.2022.969678 36466911 PMC9716460

[66] Wang X Zhang H Wang Y Bramasole L Guo K Mourtada F. DNA sensing via the cGAS/STING pathway activates the immunoproteasome and adaptive T-cell immunity. EMBO J. (2023) 42:e110597. doi: 10.15252/embj.2022110597 36912165 PMC10106989

[67] Bakhoum SF Ngo B Laughney AM Cavallo JA Murphy CJ Ly P. Chromosomal instability drives metastasis through a cytosolic DNA response. Nature. (2018) 553:467–72. doi: 10.1038/nature25432 PMC5785464 29342134

[68] Drews RM Hernando B Tarabichi M Haase K Lesluyes T Smith PS. A pan-cancer compendium of chromosomal instability. Nature. (2022) 606:976–83. doi: 10.1038/s41586-022-04789-9 PMC7613102 35705807

[69] Hong C Schubert M Tijhuis AE Requesens M Roorda M van den Brink A. cGAS-STING drives the IL-6-dependent survival of chromosomally instable cancers. Nature. (2022) 607:366–73. doi: 10.1038/s41586-022-04847-2 35705809

[70] Beernaert B Parkes EE. cGAS-STING signalling in cancer: striking a balance with chromosomal instability. Biochem Soc Trans. (2023) 51:539–55. doi: 10.1042/bst20220838 PMC10212549 36876871

[71] Chen YH Chen HH Wang WJ Chen HY Huang WS Kao CH. TRABID inhibition activates cGAS/STING-mediated anti-tumor immunity through mitosis and autophagy dysregulation. Nat Commun. (2023) 14:3050. doi: 10.1038/s41467-023-38784-z 37237031 PMC10220035

[72] Yang C Liang Y Liu N Sun M. Role of the cGAS-STING pathway in radiotherapy for non-small cell lung cancer. Radiat Oncol. (2023) 18:145. doi: 10.1186/s13014-023-02335-z 37667279 PMC10478265

[73] Yang Y Wu M Cao D Yang C Jin J Wu L. ZBP1-MLKL necroptotic signaling potentiates radiation-induced antitumor immunity via intratumoral STING pathway activation. Sci Adv. (2021) 7:eabf6290. doi: 10.1126/sciadv.abf6290 34613770 PMC8494295

[74] Deng L Liang H Xu M Yang X Burnette B Arina A. STING-dependent cytosolic DNA sensing promotes radiation-induced type I interferon-dependent antitumor immunity in immunogenic tumors. Immunity. (2014) 41:843–52. doi: 10.1016/j.immuni.2014.10.019 PMC5155593 25517616

[75] Gan Y Li X Han S Liang Q Ma X Rong P. The cGAS/STING Pathway: A novel target for cancer therapy. Front Immunol. (2021) 12:795401. doi: 10.3389/fimmu.2021.795401 35046953 PMC8761794

[76] Garland KM Sheehy TL Wilson JT. Chemical and biomolecular strategies for STING pathway activation in cancer immunotherapy. Chem Rev. (2022) 122:5977–6039. doi: 10.1021/acs.chemrev.1c00750 35107989 PMC8994686

[77] Zhou L Zhang YF Yang FH Mao HQ Chen Z Zhang L. Mitochondrial DNA leakage induces odontoblast inflammation via the cGAS-STING pathway. Cell Commun Signal. (2021) 19:58. doi: 10.1186/s12964-021-00738-7 34016129 PMC8136190

[78] Cheng AN Cheng LC Kuo CL Lo YK Chou HY Chen CH. Mitochondrial Lon-induced mtDNA leakage contributes to PD-L1-mediated immunoescape via STING-IFN signaling and extracellular vesicles. J Immunother Cancer. (2020) 8:e001372. doi: 10.1136/jitc-2020-001372 33268351 PMC7713199

[79] Heyman M Grandér D Bröndum-Nielsen K Liu Y Söderhäll S Einhorn S. Deletions of the short arm of chromosome 9, including the interferon-alpha/-beta genes, in acute lymphocytic leukemia. Studies on loss of heterozygosity, parental origin of deleted genes and prognosis. Int J Cancer. (1993) 54:748–53. doi: 10.1002/ijc.2910540507 8100807

[80] Xia T Konno H Ahn J Barber GN. Deregulation of STING signaling in colorectal carcinoma constrains DNA damage responses and correlates with tumorigenesis. Cell Rep. (2016) 14:282–97. doi: 10.1016/j.celrep.2015.12.029 PMC4845097 26748708

[81] Ablasser A Schmid-Burgk JL Hemmerling I Horvath GL Schmidt T Latz E. Cell intrinsic immunity spreads to bystander cells via the intercellular transfer of cGAMP. Nature. (2013) 503:530–4. doi: 10.1038/nature12640 PMC4142317 24077100

[82] Luteijn RD Zaver SA Gowen BG Wyman SK Garelis NE Onia L. SLC19A1 transports immunoreactive cyclic dinucleotides. Nature. (2019) 573:434–8. doi: 10.1038/s41586-019-1553-0 PMC6785039 31511694

[83] Chen Q Boire A Jin X Valiente M Er EE Lopez-Soto A. Carcinoma-astrocyte gap junctions promote brain metastasis by cGAMP transfer. Nature. (2016) 533:493–8. doi: 10.1038/nature18268 PMC5021195 27225120

[84] Kitai Y Kawasaki T Sueyoshi T Kobiyama K Ishii KJ Zou J. DNA-containing exosomes derived from cancer cells treated with topotecan activate a STING-dependent pathway and reinforce antitumor immunity. J Immunol. (2017) 198:1649–59. doi: 10.4049/jimmunol.1601694 28069806

[85] Woo SR Fuertes MB Corrales L Spranger S Furdyna MJ. Leung MY et al: STING-dependent cytosolic DNA sensing mediates innate immune recognition of immunogenic tumors. Immunity. (2014) 41:830–42. doi: 10.1016/j.immuni.2014.10.017 PMC4384884 25517615

[86] Yi M Niu M Zhang J Li S Zhu S Yan Y. Combine and conquer: manganese synergizing anti-TGF-β/PD-L1 bispecific antibody YM101 to overcome immunotherapy resistance in non-inflamed cancers. J Hematol Oncol. (2021) 14:146. doi: 10.1186/s13045-021-01155-6 34526097 PMC8442312

[87] Zhang R Wang C Guan Y Wei X Sha M Yi M. Manganese salts function as potent adjuvants. Cell Mol Immunol. (2021) 18:1222–34. doi: 10.1038/s41423-021-00669-w PMC8093200 33767434

[88] Song Y Liu Y Teo HY Hanafi ZB Mei Y Zhu Y. Manganese enhances the antitumor function of CD8(+) T cells by inducing type I interferon production. Cell Mol Immunol. (2021) 18:1571–4. doi: 10.1038/s41423-020-00524-4 PMC8166851 32801367

[89] Marcus A Mao AJ Lensink-Vasan M Wang L Vance RE Raulet DH. Tumor-derived cGAMP triggers a STING-mediated interferon response in non-tumor cells to activate the NK cell response. Immunity. (2018) 49:754–763.e754. doi: 10.1016/j.immuni.2018.09.016 30332631 PMC6488306

[90] Bakhoum SF Cantley LC. The multifaceted role of chromosomal instability in cancer and its microenvironment. Cell. (2018) 174:1347–60. doi: 10.1016/j.cell.2018.08.027 PMC6136429 30193109

[91] Ou L Zhang A Cheng Y Chen Y. The cGAS-STING pathway: A promising immunotherapy target. Front Immunol. (2021) 12:795048. doi: 10.3389/fimmu.2021.795048 34956229 PMC8695770

[92] Shih AY Damm-Ganamet KL Mirzadegan T. Dynamic structural differences between human and mouse STING lead to differing sensitivity to DMXAA. Biophys J. (2018) 114:32–9. doi: 10.1016/j.bpj.2017.10.027 PMC5773749 29320694

[93] Papaevangelou E Esteves AM Dasgupta P Galustian C. Cyto-IL-15 synergizes with the STING agonist ADU-S100 to eliminate prostate tumors and confer durable immunity in mouse models. Front Immunol. (2023) 14:1196829. doi: 10.3389/fimmu.2023.1196829 37465665 PMC10350564

[94] Esteves AM Papaevangelou E Dasgupta P Galustian C. Combination of interleukin-15 with a STING agonist, ADU-S100 analog: A potential immunotherapy for prostate cancer. Front Oncol. (2021) 11:621550. doi: 10.3389/fonc.2021.621550 33777767 PMC7988118

[95] Luo J Pang S Hui Z Zhao H Xu S Yu W. Blocking Tim-3 enhances the anti-tumor immunity of STING agonist ADU-S100 by unleashing CD4(+) T cells through regulating type 2 conventional dendritic cells. Theranostics. (2023) 13:4836–57. doi: 10.7150/thno.86792 PMC10526657 37771774

[96] Meric-Bernstam F Sweis RF Hodi FS Messersmith WA Andtbacka RHI Ingham M. Phase I dose-escalation trial of MIW815 (ADU-S100), an intratumoral STING agonist, in patients with advanced/metastatic solid tumors or lymphomas. Clin Cancer Res. (2022) 28:677–88. doi: 10.1158/1078-0432.Ccr-21-1963 34716197

[97] Meric-Bernstam F Sweis RF Kasper S Hamid O Bhatia S Dummer R. Combination of the STING agonist MIW815 (ADU-S100) and PD-1 inhibitor spartalizumab in advanced/metastatic solid tumors or lymphomas: an open-label, multicenter, phase ib study. Clin Cancer Res. (2023) 29:110–21. doi: 10.1158/1078-0432.Ccr-22-2235 PMC11188043 36282874

[98] Chang W Altman MD Lesburg CA Perera SA Piesvaux JA Schroeder GK. Discovery of MK-1454: A potent cyclic dinucleotide stimulator of interferon genes agonist for the treatment of cancer. J Med Chem. (2022) 65:5675–89. doi: 10.1021/acs.jmedchem.1c02197 35332774

[99] McIntosh JA Liu Z Andresen BM Marzijarani NS Moore JC Marshall NM. A kinase-cGAS cascade to synthesize a therapeutic STING activator. Nature. (2022) 603:439–44. doi: 10.1038/s41586-022-04422-9 35296845

[100] Smith M Chin D Chan S Mahady S Campion L Morgan C. Abstract 5567: *In vivo* administration of the STING agonist, JNJ-67544412, leads to complete regression of established murine subcutaneous tumors. Cancer Res. (2020) 80:5567–7. doi: 10.1158/1538-7445.AM2020-5567

[101] Challa S Zhou S Sheri A Padmanabhan S Delaney S Meher G. Abstract B40: Nucleotide analogs as novel STING agonists for immuno-oncology. Cancer Immunol Res. (2017) 5:B40–0. doi: 10.1158/2326-6074.TUMIMM16-B40

[102] ABBAS A Strauss J Janku F Karim R Olszanski A Luke J. P01.01 A Phase 1a/1b dose-escalation study of intravenously administered SB 11285 alone and in combination with nivolumab in patients with advanced solid tumors. J ImmunoTherapy Cancer. (2020) 8:A7–8. doi: 10.1136/jitc-2020-ITOC7.14

[103] Sun X Zhang Y Li J Park KS Han K Zhou X. Amplifying STING activation by cyclic dinucleotide-manganese particles for local and systemic cancer metalloimmunotherapy. Nat Nanotechnol. (2021) 16:1260–70. doi: 10.1038/s41565-021-00962-9 PMC8595610 34594005

[104] Amouzegar A Chelvanambi M Filderman JN Storkus WJ Luke JJ. STING agonists as cancer therapeutics. Cancers (Basel). (2021) 13:2695. doi: 10.3390/cancers13112695 34070756 PMC8198217

[105] Wang J Falchook G Nabhan S Kulkarni M Sandy P Dosunmu O. 495 Trial of SNX281, a systemically delivered small molecule STING agonist, in solid tumors and lymphomas. J ImmunoTherapy Cancer. (2021) 9:A527–7. doi: 10.1136/jitc-2021-SITC2021.495

[106] Ding C Song Z Shen A Chen T Zhang A. Small molecules targeting the innate immune cGAS−STING−TBK1 signaling pathway. Acta Pharm Sin B. (2020) 10:2272–98. doi: 10.1016/j.apsb.2020.03.001 PMC7745059 33354501

[107] Banerjee M Basu S Middya S Shrivastava R Ghosh R Pryde DC. Abstract LB-061: CRD5500: A versatile small molecule STING agonist amenable to bioconjugation as an ADC. Cancer Res. (2019) 79:LB–061-LB-061. doi: 10.1158/1538-7445.AM2019-LB-061

[108] Wang Z Dove P Rosa D Bossen B Helke S Charbonneau M. Abstract 3854: Preclinical characterization of a novel non-cyclic dinucleotide small molecule STING agonist with potent antitumor activity in mice. Cancer Res. (2019) 79:3854–4. doi: 10.1158/1538-7445.AM2019-3854

[109] Jekle A Thatikonda SK Jaisinghani R Ren S Kinkade A Stevens SK. Tumor regression upon intratumoral and subcutaneous dosing of the STING agonist ALG-031048 in mouse efficacy models. Int J Mol Sci. (2023) 24:16274. doi: 10.3390/ijms242216274 38003463 PMC10671074

[110] Pan BS Perera SA Piesvaux JA Presland JP Schroeder GK Cumming JN. An orally available non-nucleotide STING agonist with antitumor activity. Science. (2020) 369:eaba6098. doi: 10.1126/science.aba6098 32820094

[111] Yi M Niu M Wu Y Ge H Jiao D Zhu S. Combination of oral STING agonist MSA-2 and anti-TGF-β/PD-L1 bispecific antibody YM101: a novel immune cocktail therapy for non-inflamed tumors. J Hematol Oncol. (2022) 15:142. doi: 10.1186/s13045-022-01363-8 36209176 PMC9548169

[112] Lin Z Wang Q Jiang T Wang W Zhao JJ. Targeting tumor-associated macrophages with STING agonism improves the antitumor efficacy of osimertinib in a mouse model of EGFR-mutant lung cancer. Front Immunol. (2023) 14:1077203. doi: 10.3389/fimmu.2023.1077203 36817465 PMC9933873

[113] Ramanjulu JM Pesiridis GS Yang J Concha N Singhaus R Zhang SY. Design of amidobenzimidazole STING receptor agonists with systemic activity. Nature. (2018) 564:439–43. doi: 10.1038/s41586-018-0705-y 30405246

[114] Chan SR Bignan G Pierson E Mahady S Ta H Schepens W. Abstract 5567A: JNJ-’6196: A next generation STING agonist with potent preclinical activity by the IV route. Cancer Res. (2020) 80:5567A–A. doi: 10.1158/1538-7445.Am2020-5567a

[115] Wu YT Fang Y Wei Q Shi H Tan H Deng Y. Tumor-targeted delivery of a STING agonist improvescancer immunotherapy. Proc Natl Acad Sci USA. (2022) 119:e2214278119. doi: 10.1073/pnas.2214278119 36442099 PMC9894229

[116] Duvall JR Thomas JD Bukhalid RA Catcott KC Bentley KW Collins SD. Discovery and optimization of a STING agonist platform for application in antibody drug conjugates. J Med Chem. (2023) 66:10715–33. doi: 10.1021/acs.jmedchem.3c00907 PMC10424177 37486969

[117] Zhou L Yi W Zhang Z Shan X Zhao Z Sun X. STING agonist-boosted mRNA immunization via intelligent design of nanovaccines for enhancing cancer immunotherapy. Natl Sci Rev. (2023) 10:nwad214. doi: 10.1093/nsr/nwad214 37693123 PMC10484175

[118] Zhang Y Shen T Zhou S Wang W Lin S Zhu G. pH-responsive STING-activating DNA nanovaccines for cancer immunotherapy. Adv Ther (Weinh). (2020) 3:2000083. doi: 10.1002/adtp.202000083 34337143 PMC8323737

[119] Gu Y Lin S Wu Y Xu P Zhu W Wang Y. Targeting STING activation by antigen-inspired mnO(2) nanovaccines optimizes tumor radiotherapy. Adv Healthc Mater. (2023) 12:e2300028. doi: 10.1002/adhm.202300028 36876892

[120] Fu J Kanne DB Leong M Glickman LH McWhirter SM Lemmens E. STING agonist formulated cancer vaccines can cure established tumors resistant to PD-1 blockade. Sci Transl Med. (2015) 7:283ra252. doi: 10.1126/scitranslmed.aaa4306 PMC4504692 25877890

[121] Su T Cheng F Qi J Zhang Y Zhou S Mei L. Responsive multivesicular polymeric nanovaccines that codeliver STING agonists and neoantigens for combination tumor immunotherapy. Adv Sci (Weinh). (2022) 9:e2201895. doi: 10.1002/advs.202201895 35712773 PMC9376841

[122] Jiang X Wang J Zheng X Liu Z Zhang X Li Y. Intratumoral administration of STING-activating nanovaccine enhances T cell immunotherapy. J Immunother Cancer. (2022) 10:e003960. doi: 10.1136/jitc-2021-003960 35623658 PMC9150169

[123] Liu H Hu Z Chen H Yan Y Le Z Wei C. Self-degradable poly(β-amino ester)s promote endosomal escape of antigen and agonist. J Control Release. (2022) 345:91–100. doi: 10.1016/j.jconrel.2022.03.006 35259460

[124] Cheng L Zhang P Liu Y Liu Z Tang J Xu L. Multifunctional hybrid exosomes enhanced cancer chemo-immunotherapy by activating the STING pathway. Biomaterials. (2023) 301:122259. doi: 10.1016/j.biomaterials.2023.122259 37531777

[125] Liu J Xiang J Jin C Ye L Wang L Gao Y. Medicinal plant-derived mtDNA via nanovesicles induces the cGAS-STING pathway to remold tumor-associated macrophages for tumor regression. J Nanobiotechnology. (2023) 21:78. doi: 10.1186/s12951-023-01835-0 36879291 PMC9990354

[126] Bao P Gu HY Ye JJ He JL Zhong Z Yu AX. Chimeric exosomes functionalized with STING activation for personalized glioblastoma immunotherapy. Adv Sci (Weinh). (2024) 11:e2306336. doi: 10.1002/advs.202306336 38072677 PMC10853748

[127] Luke JJ Piha-Paul SA Medina T Verschraegen CF Varterasian M Brennan AM. Phase I Study of SYNB1891, an Engineered E. coli Nissle Strain Expressing STING Agonist, with and without Atezolizumab in Advanced Malignancies. Clin Cancer Res. (2023) 29:2435–44. doi: 10.1158/1078-0432.Ccr-23-0118 PMC11225568 37227176

[128] Zhao K Huang J Zhao Y Wang S Xu J Yin K. Targeting STING in cancer: Challenges and emerging opportunities. Biochim Biophys Acta Rev Cancer. (2023) 1878:188983. doi: 10.1016/j.bbcan.2023.188983 37717857

[129] Wang Q Bergholz JS Ding L Lin Z Kabraji SK Hughes ME. STING agonism reprograms tumor-associated macrophages and overcomes resistance to PARP inhibition in BRCA1-deficient models of breast cancer. Nat Commun. (2022) 13:3022. doi: 10.1038/s41467-022-30568-1 35641483 PMC9156717

[130] Ma Z Li Z Mao Y Ye J Liu Z Wang Y. AhR diminishes the efficacy of chemotherapy via suppressing STING dependent type-I interferon in bladder cancer. Nat Commun. (2023) 14:5415. doi: 10.1038/s41467-023-41218-5 37670034 PMC10480448

